# Examining the Walking Accessibility, Willingness, and Travel Conditions of Residents in Saudi Cities

**DOI:** 10.3390/ijerph16040545

**Published:** 2019-02-14

**Authors:** Muhammad Tauhidur Rahman, Kh. Md. Nahiduzzaman

**Affiliations:** 1 Department of City and Regional Planning, King Fahd University of Petroleum and Minerals, KFUPM Box 5053, Dhahran 31261, Saudi Arabia; 2 School of Engineering, The University of British Columbia (UBC)—Okanagan, 1137 Alumni Ave, Kelowna 1V1 1V7, BC, Canada; nahid@kfupm.edu.sa

**Keywords:** walkability, built environment, accessibility, willingness and perception, safety, road conditions

## Abstract

Rapid urban expansion and population growth in Saudi cities over the past four decades have increased vehicular accidents and traffic congestion and have impacted the daily walking conditions of the residents. Walking has various health and environmental benefits. In North American and European countries, three factors have been found to motivate a resident to walk within their community: their accessibility to community social and business facilities, their perception and willingness, and the safety conditions of the roads and sidewalks within their community for walking. This study examined these factors and their role in the walking habits of the residents in the neighborhoods of Doha and Dana districts in Saudi Arabia’s eastern city of Dhahran. Data were collected through field observations and by randomly sampling and interviewing 200 residents. Geographic Information Systems (GIS) and SPSS statistical software were used for data analysis. The results show that most of the community facilities are randomly placed in the districts. Mosques are the closest facility to each resident with an average accessibility distance of 242m. Almost 43% of the respondents prefer daily walking while the rest are hesitant due to hot weather during summer and narrow and poorly designed sidewalks. The sidewalks were also found to be blocked by trees, street signals, and illegally parked vehicles. Future studies should explore the accessibility to facilities, willingness, climate, and health conditions of the residents, and the road and sidewalk conditions for walking in other cities of the Kingdom.

## 1. Introduction

Every day, millions of adults and children in both developed and developing countries are diagnosed with obesity-related health issues including diabetes, cardiovascular diseases, and cancer [1,2,3,4]. For them, a simple but daily 30 minutes of physical activity, such as walking, can be a path to reducing or managing body weight and obesity related co-morbidities, as well as, living a long and healthy life [5]. Walking has economic and environmental benefits as well. By reducing the trips made in private vehicles, residents see a decline in their monthly expenses for gasoline and maintenance of their vehicles and also lessen the vehicle emissions and air pollutions within their city [6,7]. Over the past 20 years, extensive studies in various major cities in the world have focused on determining the reasons and factors that increase a person’s willingness to walk for its health benefits, recreation, and as a mode of transportation to reach destinations and fulfill his or her basic needs within the local community [8,9,10,11]. 

While numerous factors have been identified in these studies, they can primarily be placed into three major categories. First, objective measures such as accessibility and close proximity, pattern, and density of facilities are some of the most crucial factors to be associated with an urban resident’s inclination for walking [8,12,13,14]. Examining the distances between a resident’s house and to the nearest service center in the city of Paris, France, [15] found that residents are more likely to walk to near facilities than service centers at distant locations irrespective of their socio-economic conditions. In San Diego [16] observed that neighborhoods with high-walkability rates had a high concentration of commercial centers (restaurants, banks, grocery and convenient stores) along the main roads of residential neighborhoods. Surveying older Australians, [11] showed that residents who lived near facilities that are close and accessible are more likely to walk and be active than distant residents. Second, subjective measures such as a person’s perception and attitude towards walking will likely impact their daily walking habits and behaviors. Surveying school children’s commuting behavior to schools in the morning, [17] concluded that the perception and attitude of the parents towards their kids walking to school was one of the main predictors of their children commuting actively. Perceptions of street connectivity and traffic safety were also found to be the factors affecting the walking behaviors of senior adults [18]. Finally, studies have linked various neighborhood street characteristics including traffic flow, the presence of green environment, and width and conditions of the sidewalk with the amount people walk within their neighborhoods [19]. While green environments with wider, cleaner, and obstacle-free sidewalks stimulated people to walk [20,21,22], the presence of crime, discontinued or lacking sidewalks, and traffic safety concerns discouraged residents from walking and biking in their communities [23,24].

Since the early 1970s, cities in the Kingdom of Saudi Arabia have increased significantly in size and experienced rapid population growth due to rising economic growth and prosperity [25,26]. Such growth of cities has had various environmental, social, and economic consequences, including air and water pollution, frequent incidence of flooding, rising land surface temperatures, lack of affordable housing, and rising energy demand [27,28,29,30,31,32]. It is also increasing the total number of vehicles in each city and affecting their transportation systems and road networks by causing more traffic congestion, frequent accidental injuries and deaths, increasing average trip time, and reducing the available parking spaces in the commercial centers and residential neighborhoods [33,34]. Thus, it has directly impacted the walking conditions of the residents in Saudi cities. However, only few studies have examined the behavior and walking conditions of the residents within these cities. [35] examined socio-cultural factors on the walking behavior of the residents in the Saudi capital city of Riyadh. Also for Riyadh, [36] analyzed the incidents and injury patterns among pedestrians and vehicle accidents between 1997 and 1999. [6] explored the difficulties that are faced by pedestrians within a small national park in the Saudi eastern coastal city of Dammam and discussed how smart solutions can be utilized to resolve some of those difficulties. Finally, [37] attempted to redesign the old souk (shopping center) of the southwestern coastal city of Jizan to improve its walking conditions and overall quality for its visitors. 

As none of the abovementioned studies have investigated the previously discussed three main factors affecting walkability in Saudi cities, this study aims to fulfill that gap. It will do so by first examining the distribution patterns of community services in the Doha and Dana residential neighborhoods within the city of Dhahran in the Eastern Province of the Kingdom. Using a detailed survey questionnaire, the study will then investigate the travel behavior, perceptions, and the resident’s willingness to walk to the community service centers. Finally, it will assess the designs and conditions of the streets and sidewalks within the two neighborhoods. In the following sections, a brief description of the study areas is given in Section 2. Section 3 highlights the detailed methodology used in the study. The results are provided in Section 4 and are discussed in Section 5. Finally, the concluding remarks and the path for future studies are provided in Section 6. 

## 2. Study Area

**Figure 1:**
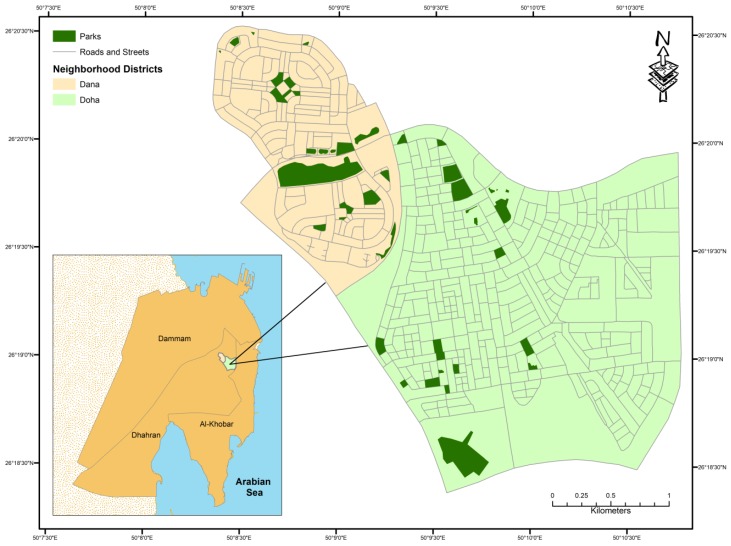
Study Area in the City of Dhahran.

The residential neighborhood districts of Doha and Dana with a total area of almost 9 sq. km within the city of Dhahran were chosen as the area of interest for this study. Dhahran, along with the cities of Al-Khobar and Dammam, are the three cities that are part of the greater Dammam Metropolitan Area in the Eastern Province of the Kingdom of Saudi Arabia (Figure 1). The population of Dhahran has increased tremendously over the past several decades after oil was discovered in the region and the establishment of the headquarters of the Arabian American Oil Company (later renamed Saudi ARAMCO) along with the King Fahd University of Petroleum and Minerals (KFUPM) in 1963. Today, the city has a population of approximately 200,000 with an annual growth rate of 6.5% [38]. 

To provide housing and daily needs of the residents in the city, several planned residential areas had been developed over the past three decades. Doha and Dana are two of such major residential communities housing mostly regular and contracted employees of Saudi Aramco along with few faculty members and students of KFUPM. While the residents are mostly Saudis, there are also expatriates residing in the communities from the neighboring Arab, south, and southeast Asian countries. The city experiences a desert climate with hot and humid summers (temperatures ranging from 21 °C–49 °C) and cool and dry (10 °C–23 °C) winters [39]. 

## 3. Data and Methods

The data for this study were collected by the authors and two research assistants in three primary phases to fulfill the three goals of the study. In the first phase, the distribution patterns of the community services were examined by utilizing GPS and Geographic Information Systems (GIS) technologies. A total of 6 separate field visits during the month of September of 2013 were made to 10 different types of individual community services and facilities (Table 1) and their latitudes and longitudes were recorded using a Trimble Juno SB portable GPS unit. The coordinates were transferred into the ArcGIS v.10 software (ESRI, Redlands, CA, USA) and using available paper maps and imagery from Google Earth, the streets and all the individual land parcels within the study area were manually digitized in the ArcGIS software. The distances between each individual parcel and the nearest facilities were then calculated. The Nearest Neighbor Index (NNI) for each type of community service facility were calculated in ArcGIS to examine their pattern of distribution by knowing if the locations of the facilities are systematically clustered together or if they are dispersed or randomly built throughout the study area. The NNI was calculated in ArcGIS using the following equation where ${\bar{D}}_{nnd}$ is the average distance between each facility and the nearest facility and ${\bar{D}}_{ran}$ is the distance that would be expected if the facilities were randomly distributed:

(1) $$NNI=\frac{{\bar{D}}_{nnd}}{{\bar{D}}_{ran}};\;\mathrm{where}\;{\bar{D}}_{nnd}=\frac{\sum_{i=1}^{n}d_{i}}{n}\;\mathrm{and}\;{\bar{D}}_{ran}=0.5\times \sqrt{\frac{A}{n}}$$

In the equations, *n* is the total number of facilities within the study area; $d_{i}$ is the distance between each facility and its nearest neighboring facility and *A* is the total area for study (8,775,460 m^2^). The NNI value near 0 indicates clustering while value close to 1 suggests the facilities are randomly distributed. Values greater than 1 suggests that the points are spatially dispersed. For facilities that had a clustering pattern, hotspot analyses using the Nearest Neighbor Hierarchical Clustering (NnH) algorithm in the CrimeSTAT v. 3.3 software platform (The National Institute of Justice, Washington D. C., WA, USA) were performed.

In the second phase, attempts were made to examine the perception and willingness of the residents of the neighborhood towards walking to gather an in-depth understanding on the nature of walkability within the neighborhoods of Doha and Dana. To do so, a detailed survey questionnaire consisting of both open ended and multiple choice questions categorized into three major groups was created. The first set of questions focused on the basic needs and requirements that are fulfilled by the respondents and the mode of transportation that is used to fulfill them. The second set of questions investigated the typical daily walking distance and frequency for the respondents who preferred walking. Finally, the reasons why the respondents who are hesitant to walk were explored. The questionnaires were completed by a total of 200 respondents (from separate households) selected through systematic random sampling method from various parts of the study areas between September and December of 2013. The data were then entered into SPSS v. 17 statistical software package (SPSS Inc., Chicago, IL, USA) for further analysis.

Finally, in the third phase, nine separate field visits were made in the months of January and February of 2014 across the study area to examine the neighborhood walking conditions. During the visits, detailed auditing of the streets and sidewalks including the presence and structural conditions of sidewalks for walking, their vacancy, and usability were noted using a standardized checklist and captured through digital photographs using a D3200 Digital Single Lens Reflex (DSLR) digital camera (Nikon, Tokyo, Japan). Width of sidewalks were also measured using a Vata 30 m fiberglass measuring tape.

## 4. Results 

### 4.1. Patterns and Distribution of Community Services

**Figure 2:**
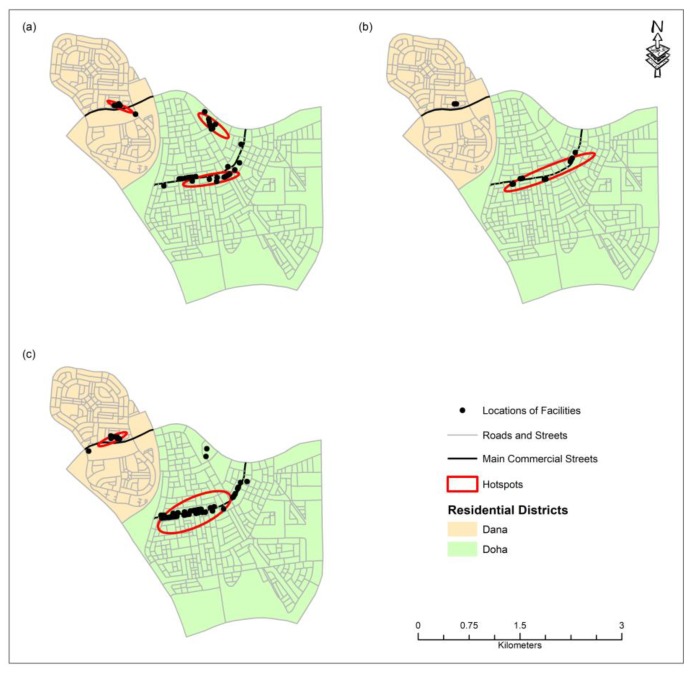
Hotspots of coffee shops and restaurants (**a**), laundry facilities (**b**), and miscellaneous commercial facilities (**c**).

**Figure 3:**
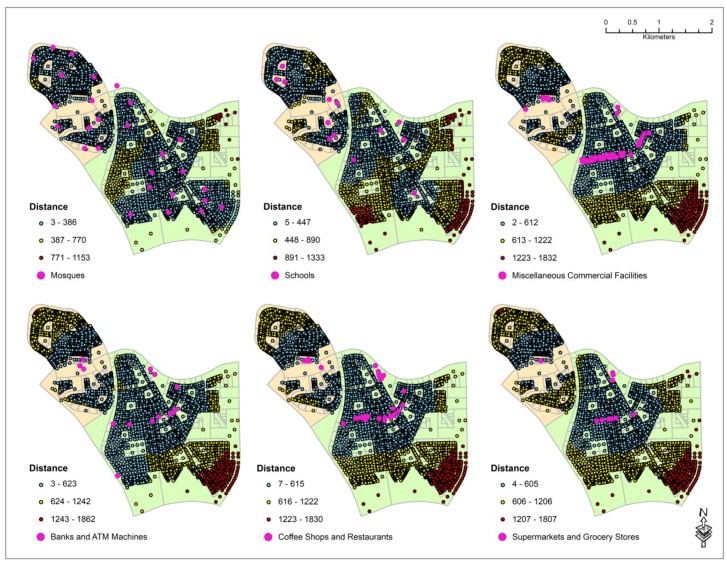
Distances in meters between the residential parcels and their nearest mosque, school, bank and ATM machine, coffee shop and restaurant, supermarket and grocery store, and salon.

**Figure 4:**
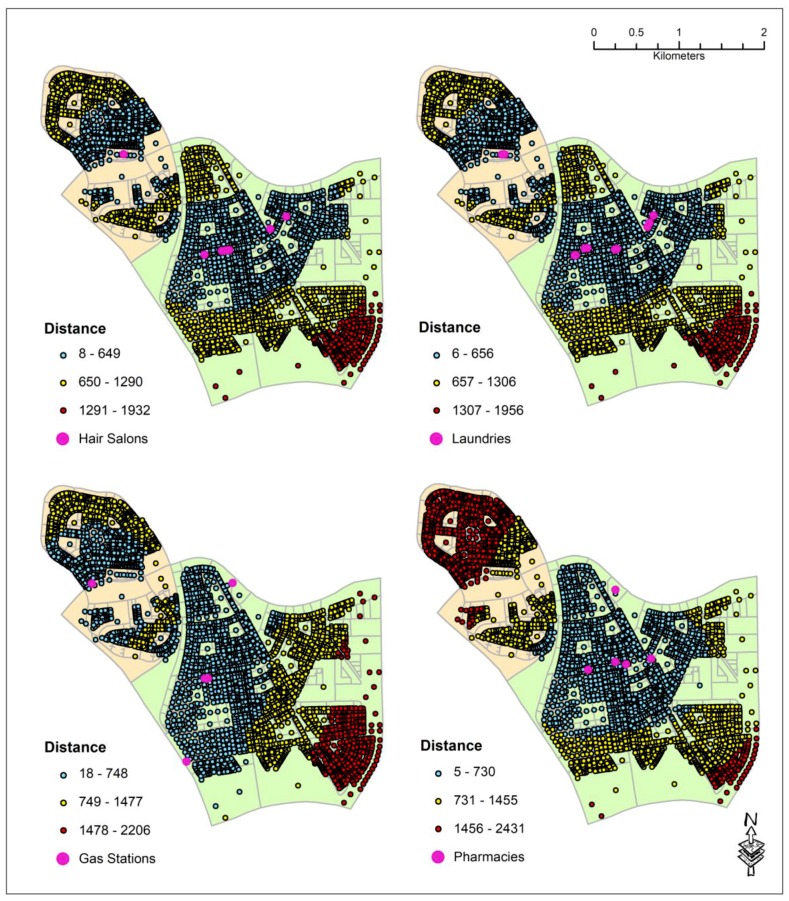
Distances in meters between the residential parcels and their nearest laundry, gas station, and pharmacy.

Based on the field surveys, it was found that the commercial and community service facilities within the two residential neighborhoods can be grouped into 10 major categories. The category of the facilities, their numbers, and their distribution type based on the NNI values are provided in Table 1. For this study, NNI values below 0.25 were considered to indicate clustering, values between 0.25 and 1 indicated random pattern, and above 1 were considered to indicate dispersed pattern. Based on the NNI values, mosques and gas stations are all dispersely located in the study area. The schools, hair salons, banks and ATMs, pharmacies, supermarkets and grocery stores are randomly established whereas coffee shops and restaurants, various miscellaneous commercial facilities, and laundry services are clustered within certain areas. Therefore, hotspot analysis using the Nearest Neighbor Hierarchical Clustering (NnH) algorithm using the parameters shown in Table 2 was performed on the latter three features. These parameters produced the logical numbers of clusters for each category of facility. Figure 2 shows the hotspots produced for each category. The distances between the centroid of each digitized residential land parcel and to the nearest facility by type were then calculated. The minimum, maximum, and the mean distances for each category of services are shown in Table 3 and their distributions are shown in Figure 3 and Figure 4.

### 4.2. Travel and Walking Behavior, and the Willingness to Walk by the Residents

Among the 200 surveyed respondents, 138 (69%) were male and 62 (31%) were female with a mean age of 30.33 (standard deviation of 11.23). The first set of questions focused on the requirements (i.e. groceries, banking, school, etc.) that are fulfilled by the respondents and the mode of transportation that is used to fulfill them. The results for these set of questions are shown in Table 4 and Table 5. Second, the typical daily walking distance and frequency for the respondents who preferred walking were investigated. Approximately 42.5% of the respondents preferred walking. Among them, 84% are male, 91.5% of them usually walk up to 1 km. daily, while the remaining 8.5% walk between 1–2 km. per day. Finally, the gender-wise reasons why the respondents are hesitant to walk were explored and the summary of their answers are provided in Table 6.

### 4.3. Neighborhood Walking Conditions

**Figure 5:**
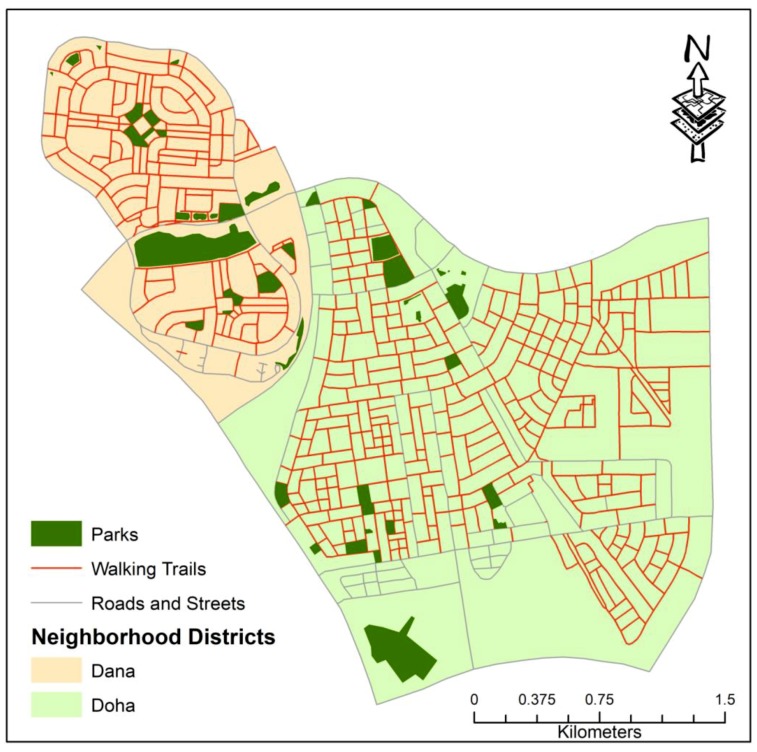
Streets with walking trails within the two residential neighborhood districts.

Results from the field survey and GIS analyses show that the two neighborhoods have a total street distance of approximately 107 km. Among them, 82.9 km (77.4%) have sidewalks or walking trails (Figure 5). However, their usability for walking is limited for several reasons. First, the sidewalks were found to be quite narrow (less than 1 meter) and poorly designed with date and palm trees along with lamp posts and street signs erected in the middle. Second, 24% of the sidewalks were seen to be occupied by parked vehicles of the surrounding residents. Almost 21% of the sidewalks have permanent constructions including walking ramps and carports. Streets and sidewalks were also found to be blocked with construction materials and hanging date leaves. These observations were also noted by the surveyed respondents (as shown in Table 6) that deter their willingness to walk. 

## 5. Discussion 

### 5.1. Patterns and Distribution of Community Services

The results reveal that mosques are the most dispersely (NNI = 1.25) located facilities within the study area. However, they are the closest (mean distance of 242 m) facilities to every single parcel. Saudi Arabia is the birthplace of Islam and praying in congregation in mosques is a major part of the religion and culture. Therefore, it is necessary and logical for mosques to be the closest features and within walking distances from each parcel. This finding also corresponds to the dispersed and decentralized distribution patterns of religious places of worship found in American and European cities [40]. Surprisingly, it was found that parcels located in the north-eastern and central-western part of the study area are quite far from the nearest mosque (Figure 3). This can be attributed to the size and boundary of the study area. It is quite possible that few mosques that are within walking distances from these parcels were not considered because of their location just outside the boundary of the study area. Therefore, further analysis needs to be conducted for not only mosques within the two districts but perhaps encompassing mosques within the entire city of Al-Khobar area to understand their true distribution pattern.

Contrary to mosques, supermarkets and grocery stores are the least randomly located commercial centers among all the features within the two districts. It was found that the average distance between parcels and the nearest supermarket is 649 m (Table 3). However, in Dana, only one small grocery store is serving the people of the entire neighborhood and since it is located almost in the center of the neighborhood, people living in the northwestern and southeastern parts of the district have to travel between 605 m and 1.2 km to reach this store. In Doha, there are 9 grocery stores, but they are very near to each other and effectively serving the residents in the central and northwestern parts of the district. Towards the eastern parts of the district, the distance between the parcels, supermarkets, and grocery stores increases slowly from 605 m to over 1.8 km (Figure 3). 

The NNI value of pharmacies shows that they are slightly more random than supermarkets and grocery stores (Table 1). On the contrary, gas stations are spread throughout the city. Due to the low number of pharmacies and gas stations (only five) within the study area, the NNI values for these features seem to be logical (Table 1). However, the locations of additional pharmacies and gas stations are necessary to accurately recognize their distribution pattern throughout the city. The results also show that pharmacies and gas stations are the farthest facilities from each parcel (Table 3). Examining by districts, it was observed that within Dana district, most of the parcels are more than 730 m away from the nearest pharmacy, but the nearest gas stations are within 748 m from each parcel (Figure 4). In Doha district, all the parcels on the western side of the main commercial street are within 813 m from the nearest pharmacy and 748 m from gas stations. Towards the eastern side of the Doha district, the nearest gas stations and pharmacies are approximately 1.5 km away. It was found that unlike mosques, there are no pharmacies or gas stations nearby in the areas surrounding the eastern border region of Doha district. This variation may be attributed to the area’s comparatively sparse and recently expanding residential housings in the past few years.

It was quite intriguing that the financial institutions such as banks and ATM machines have the same NNI values (0.56) as that of the hair salons although the former is numbered almost twice the latter (14 vs. 8). In terms of distance from each parcel, both categories of features are within an average of 627 m (Table 3). It was quite interesting that parcels in the northwestern corner of the Dana district are more than 623 m from both Banks and ATM machines and 649 m from hair salons. This phenomenon can be due to the salons and ATM machines in Dana district being inside a mini-mall located along the main street running through the center of the district. Similar situation was also observed in Doha district. The banks and ATM machines, as well as salons, are also situated along the main commercial street of the district. Therefore, as residents move farther away from the main street towards the southeastern parts of the district, the distances between these facilities and each parcel increases gradually from 623 m to almost 1.9 km (Figure 3 and Figure 4). 

Among all the random patterned facilities, schools have the highest NNI value (0.77) since eight out of the 13 schools are in Dana district although Doha is more than three times larger in area than Dana. This is due to Dana being closer to the Saudi ARAMCO headquarters and schools are built in Dana and the adjacent parts of the eastern Doha district so that the children of the ARAMCO employees can attend the schools by walking or biking in these neighborhoods due to their short distances (within 448 m as shown in Figure 3). In the eastern parts of the Doha district, only one school is present causing the children of these neighborhoods to travel between 448 m to 1.3 km. It is difficult for these children to walk or ride their bicycles and they most likely commute by private vehicles or school buses. Due to the rising population and housing densities in this part of the district, newer schools are planned to be built within the next couple of years. 

Coffee shops and restaurants, laundry facilities, and miscellaneous commercial facilities are all clustered in the study area with an average distance of 614 m from each parcel (Table 3). However, the NNI values of these facilities indicate that laundries are more clustered than miscellaneous commercial centers, while the latter is more clustered than coffee shops and restaurants (Table 1). In Dana, coffee shops, restaurants, and miscellaneous commercial centers are all clustered in the center of the district while in Doha district, they are clustered along the main street (Figure 2). It is interesting to note that in Dana district where coffee shops, restaurants, and laundry facilities are located in a central mall, majority of the residents must travel more than 600 m to reach them whereas in Doha district where these service facilities are elongated along the main street, residents living near the southeastern part of the district must travel more than 1.2 km to reach them (Figure 3 and Figure 4). This finding suggests that a central location is more convenient for the residents than the elongated location along the main street. This is probably true for a small district. However, as the district size increases, none of the central or elongated locations are convenient for the residents. Instead, several clusters of services scattered over the larger geographical area may be more convenient for the residents for walking. 

### 5.2. Walking Behavior and Willingness to Walk by the Residents

When examining the walking pattern of the residents, this study found that although the male population is known as the main actors in fulfilling the daily needs, females are also responsible and active in carrying out some of the daily needs. Along with the male members of the households, women are taking care of the groceries, banking needs, and dropping of the children to school daily. Previous studies have found that females are more proficient, punctual, and organized when it comes to fulfilling these duties [41]. It should be noted that prior to June, 2018, women were not allowed to drive in the Kingdom and they were driven around by their male relatives (father, husband, brothers, or son) or the family driver due to the Kingdom’s custom and regulations to fulfill their daily needs. It is expected that 3 million Saudi women will obtain driver licenses by 2020 and will have a large impact on the traffic and travelling conditions of the roads in the Kingdom. It will also have a high impact on the walking conditions as well. 

When it comes to age and carrying out daily basic needs, the young teenagers, and residents between the age of 21-30 years old are the most active among all the age groups. The teens and the middle-aged (31-50) population are also engaged in various daily activities which require them to use different modes of transportation during different hours of the day and different days of the week. Therefore, it is necessary to explore what portion of the daily need-based transportation is made on foot as opposed to by motorized and non-motorized vehicles.

In terms of various transportation modes used by the residents, private vehicles ranked the highest with 90% of the residents using private vehicles to fulfill their daily activities. Almost 10% of the respondents use public transportation and bicycles for traveling to work, schools, and to buy groceries. This percentage of public transportation and bicycle use is significantly lower than European cities (i.e. Amsterdam and Copenhagen) but is at par with American cities where an average of almost 5% of few city commute by biking [42,43]. 

The study found that approximately 60% of the residents walk to their nearest facilities and almost 65% walk for recreation and health benefits. While residents of Canadian cities had a similar percentage (70%) of urban walkers, it is significantly higher when compared to American cities as only 3.4% of the Americans walk to schools or their place of employment [44,45]. Most of these pedestrians walk in the study area during winter seasons since being in a desert climate (where daily average temperatures reach almost 50 °C with very high humidity during the summer months), walking can be difficult and even hazardous for the health of the residents who can suffer from heat exhaustion and heat strokes. While hot weather limited walkability in this study’s neighborhoods, it is cold temperature, snow, and rain that has been found to modify and reduce walkability in previous studies [46,47]. The hot and humid weather was also the number one reason residents were hesitant to walk during the summer months (Table 6). However, Saudi cities have a large expatriate population and most them are low-income laborers who cannot afford private vehicles. For them, walking is a necessity rather than a choice and they are forced to walk throughout the year. Previous studies have found a similar group of residents in cities of other developed countries as well [45]. However, unlike these other cities where low-income residents had a walking safety concern in their neighborhoods due to crimes and violence [48], Saudi cities have very few crimes and being safe from crimes was not one of the cause that hindered residents from walking. The attitude and walking behaviors of the residents were also gendered dependent where males were found to be more inclined to walk on the streets when compared to the women respondents. Similar results were also found by [35] for the city of Riyadh. They found that women prefer walking in indoor facilities such as grocery stores and shopping malls whereas men walk on urban streets. Also, due to the conservative nature of the Islamic culture, women’s presence on the streets for walking is limited during certain times of the day [35]. 

### 5.3. Neighborhood Walking Conditions

The results from the field survey and GIS analysis show that approximately 77% of the streets in the study area are designed with sidewalks and walking trails (Figure 5). However, it is often difficult for the pedestrians to utilize these streets due to narrow designs, blockades by trees and street signals, and illegal parking of private and commercial vehicles. The General Theory of Walkability proposed by Jeff Speck highlights that walkable streets should fulfill four primary conditions: be useful, safe, comfortable, and interesting [49]. The streets in the study area were created to be useful. However, safety, comfort level, and aesthetic designs were not considered when they were designed and built. Recent studies have found inequalities in the street designs of American cities and pedestrians who are minorities, above 65 years old, uninsured, and members of low-income households are at high risk of being killed from car accidents while walking [50]. This study did not find such inequalities but rather streets were poorly designed throughout the study area irrespective of the socio-economic conditions of the surrounding residents. Unlike European cities that try to provide a high level of comfort for their pedestrians by providing them with benches for sitting, shading for the rain and summer heat, and reducing noise levels, such facilities are non-existent in the streets of the study area [51]. 

Based on the above-mentioned problems and widespread poor street designing for pedestrians in all parts of the residential neighborhoods within the study area, several recommendations could be made to the Dhahran municipality for their improvements. First, the city municipality needs to regularly monitor the city neighborhoods to find vehicles parked off-street on driveways and sidewalks and possibly penalize the drivers for such behaviors. The authorities also need to take a strong role and all necessary actions to encourage drivers for on-street parking and making public spaces available for daily pedestrians. Having more on-street vehicles will reduce road widths and create a physical separation between moving vehicles and walking pedestrians. They are also expected to reduce the moving vehicle speeds and traffic volumes in the residential neighborhood streets. Second, utility poles and trees that are placed and planted in the middle of the sidewalks should be removed and relocated so that they do not block the sidewalks. Utility poles can perhaps also be eliminated by placing utility power lines underground (where feasible) and their densities can be reduced. A minimum distance (i.e. 1.5 m as required for some American cities) should be maintained when planting new trees next to sidewalks. They should also be carefully placed so that they do not obstruct the views of the drivers in the streets and not enabling them to see the pedestrians on the sidewalks. Perhaps planting strips can even be introduced within the residential neighborhoods to create and improve the aesthetical views of the streets. Third, it was observed that in various locations, sidewalks were discontinuous with visible bare sands and dirt which are littered with wastes. The municipality should make sure these areas are cleaned and continued paved surfaces are created to make walking easier for the pedestrians. Fourth, traffic signals and lights along with speed bumps or roundabouts should be installed and introduced in busy intersections to reduce the vehicle speeds and avoid an accidental collision with other moving cars and pedestrians. Finally, the municipality and the regional government should initiate awareness programs to educate the residents about the benefits of walking and encourage them to make it part of their daily habit. 

### 5.4. Study Limitations

As in all studies, there are several limitations to this study. First, this study area was limited to only within two residential neighborhoods in the city of Dhahran. Distribution of facilities, perceptions of residents towards walking, and walking conditions in other neighborhoods of Dammam metropolitan area should also be investigated. Second, the impacts of climate are not considered in this study. While it is known that summer heat is a major factor that hinders people from walking in the summer, the actual temperature and humidity above which people are hesitant or feel discomfort should be examined. Third, the physical conditions of the respondents were not measured and correlated with their responses. The relationship between the weight, height, presence of heart diseases and diabetes, and other health issues with walking perception and behavior should be investigated. Fourth, the study did not consider the nationality of the residents. The perception and walking behavior of expatriate residents are expected to be different when compared with the Saudi nationals as evidenced in other GCC countries [52]. Finally, the walking behavior of the residents was not explored in relation to the distances to the nearest facilities. For example, it did not explore whether having a mosque rather than a laundry closer to home affect a person’s walking behavior. Also, the study did not explore whether the structural quality of the streets and sidewalks had any particular geographical distribution patterns (i.e. random or clustering) in the two neighborhoods.

## 6. Conclusions 

This study has examined the distribution patterns and accessibility to community facilities and services by the residents of two neighborhoods in the eastern coastal city of Dhahran, Saudi Arabia. It also explored the travel behavior and the willingness to walk by the residents in the neighborhoods. Finally, the conditions and safety concerns for walking along the sidewalks and major streets are explored. The results show that most of the community facilities are randomly placed in the study area and while mosques are the closest facility to each residential land parcel, gas stations and pharmacies are the furthest. The results also show that almost half the sampled respondents are willing to walk on daily basis while the rest are hesitant to do so due to the hot weather during summer and poorly designed sidewalks. Finally, the study found that the streets in the neighborhoods are not suitable for walking due to them being too narrow and blocked by trees, street signals, and illegally parked private and commercial vehicles. The government and city officials should implement rules and regulations to reduce these blockages to make the streets safer and pedestrian friendly. Future studies should investigate the distance and impacts of various facilities on the walking behavior of the residents in the two neighborhoods. Further studies should explore the distribution pattern and accessibility to facilities, physical conditions of the residents and their willingness to walk, and the conditions of the streets in other parts of Dammam metropolitan area as well as in other cities of the Kingdom.

## Figures and Tables

**Table 1:** The community facilities considered in the study and their distribution pattern based on the NNI values.

Type of Facility	Number of Facility	Observed Mean Distance (m)	Mean Random Distance (m)	NNI	Distribution Pattern
Laundries	9	39.17	493.72	0.08	Clustered
Misc. Commercial Facilities	71	30.97	175.78	0.18	Clustered
Coffee Shops & Restaurants	38	57.37	240.28	0.24	Clustered
Supermarket & Grocery Stores	10	228.10	468.39	0.49	Random
Pharmacies	5	362.82	662.40	0.55	Random
Banks & ATMs	14	221.50	395.86	0.56	Random
Hair Salons	8	294.26	523.67	0.56	Random
Schools	13	315.56	410.80	0.77	Random
Gas Stations	5	771.56	662.40	1.16	Dispersed
Mosques	27	357.15	285.05	1.25	Dispersed

**Table 2:** The ideal parameters (threshold distance and the minimum number of facility per hotspot) considered for hotspot analysis.

Type of Facility	Threshold Distance (m)	Minimum Number of Facility per Hotspot	Number of Hotspots Produced	Number of Facilities per Hotspot
Coffee Shops & Restaurants	500	5	3	8, 5, and 10
Laundries	1000	5	1	7
Miscellaneous Commercial Facilities	1000	5	2	7 and 50

**Table 3:** Statistics about the distances between the residential parcels and the nearest facility.

Type of Facility	Statistics of Distances (in meters)			
	Minimum	Maximum	Mean	Standard Deviation
Mosques	3	1153	242	140
Schools	5	1333	430	240
Miscellaneous Commercial Facilities	2	1832	576	383
Banks & ATMs	3	1862	590	368
Coffee Shops & Restaurants	7	1830	596	380
Supermarket & Grocery Stores	4	1807	649	356
Hair Salons	8	1932	663	378
Laundries	6	1956	670	383
Gas Stations	18	2206	807	458
Pharmacies	5	2431	963	607

**Table 4:** Distribution of the percentage of sampled residents (stratified by gender and age) who are required to fulfill the daily basic necessary needs.

Basic Needs Classification	Gender		Age in Years					
	Male *n* = 138	Female *n* = 62	<20 *n* = 28	21-30 *n* = 98	31-40 *n* = 25	41-50 *n* = 30	51-60 *n* = 9	>60 *n* = 10
Grocery	83.33	69.35	82.1	90.8	84.0	70.0	11.1	30.0
Recreation	71.01	38.71	60.7	78.6	64.0	36.7	0.0	10.0
Banking	31.16	11.29	17.9	26.5	44.0	23.3	0.0	10.0
School & Employment	40.58	22.58	53.6	37.8	24.0	30.0	11.1	20.0
Others	43.48	27.42	42.9	36.7	32.0	43.3	22.2	60.0

**Table 5:** Mode of transportation to fulfill the basic necessary needs.

Basic Needs Classification	Mode of Transport (Percentage of Total Sampled Respondents)			
	Private Vehicle	Public Transport	Biking	Walking
Grocery	95.5	9.1	9.1	59.1
Recreation	94.1	0	11.8	64.7
Banking	100	0	0	57.1
School & Employment	88.9	11.1	11.1	66.7

**Table 6:** Gender wise distribution of the percentage of total respondents and their reasons for not willing to walk.

Reason for not Willing to Walk	Gender	
	Male	Female
Hot and humid weather	36.3	32.4
Poorly designed sidewalk	15.6	8.7
Sidewalk is discontinuous	14.2	20.2
Unsafe road crossing	15.9	16.8
Design not suitable for walking	8.4	0
Narrow sidewalk	0	12.7
Sidewalk occupied by other establishments	9.5	9.2

## References

[1] De Pergola G., Silvestris F. (2013). Obesity as a major risk factor for cancer. J. Obes..

[2] Calle E.E., Thun M.J. (2004). Obesity and cancer. Oncogene.

[3] Lavie C.J., Milani R.V., Ventura H.O. (2009). Obesity and Cardiovascular Disease. Risk Factor, Paradox, and Impact of Weight Loss. J. Am. Coll. Cardiol..

[4] Al-Goblan A.S., Al-Alfi M.A., Khan M.Z. (2014). Mechanism linking diabetes mellitus and obesity. Diabetes Metab. Syndr. Obes. Targets Ther..

[5] Boston G. The Many Benefits of Walking 30 Minutes a Day. https://www.washingtonpost.com/lifestyle/wellness/the-many-benefits-of-walking-30-minutes-a-day/2015/10/19/cf12c938-71e1-11e5-9cbb-790369643cf9_story.html?utm_term=.5064ea52b381.

[6] Atef Elhamy Kamel M. (2013). Encouraging walkability in GCC cities: Smart urban solutions. Smart Sustain. Built Environ..

[7] Litman T.A. (2003). Economic Value of Walkability. Transp. Res. Rec..

[8] Humpel N. (2002). Environmental factors associated with adults’ participation in physical activity A review. Am. J. Prev. Med..

[9] Butler G.P., Orpana H.M., Wiens A.J. (2007). By your own two feet. Factors associated with active transportation in Canada. Can. J. Public Heal..

[10] Owen N., Humpel N., Leslie E., Bauman A., Sallis J.F. (2004). Understanding environmental influences on walking: Review and research agenda. Am. J. Prev. Med..

[11] Booth M.L., Owen N., Bauman A., Clavisi O., Leslie E. (2000). Social-cognitive and perceived environment influences associated with physical activity in older Australians. Prev. Med..

[12] Saelens B.E., Handy S.L. (2008). Built environment correlates of walking: A review. Med. Sci. Sports Exerc..

[13] Handy S.L., Boarnet M.G., Ewing R., Killingsworth R.E. (2002). How the built environment affects physical activity: Views from urban planning. Am. J. Prev. Med..

[14] McCormack G., Giles-Corti B., Lange A., Smith T., Martin K., Pikora T. (2004). An update of recent evidence of the relationship between objective and self-report measures of the physical environment and physical activity behaviours. J. Sci. Med. Sport.

[15] Charreire H., Weber C., Chaix B., Salze P., Casey R., Banos A., Badariotti D., Kesse-Guyot E., Hercberg S., Simon C. (2012). Identifying built environmental patterns using cluster analysis and GIS: Relationships with walking, cycling and body mass index in French adults. Int. J. Behav. Nutr. Phys. Act..

[16] Saelens B.E., Sallis J.F., Black J.B., Chen D. (2003). Neighborhood-Based Differences in Physical Activity: An Environment Scale Evaluation. Am. J. Public Health.

[17] Panter J.R., Jones A.P., van Sluijs E.M.F., Griffin S.J. (2010). Europe PMC Funders Group Attitudes, social support and environmental perceptions as predictors of active commuting behaviour in school children. J. Epidemiol. Community Health.

[18] Maisel J.L. (2016). Impact of Older Adults’ Neighborhood Perceptions on Walking Behavior. J. Aging Phys. Act..

[19] Chiang Y.C., Sullivan W., Larsen L. (2017). Measuring neighborhood walkable environments: A comparison of three approaches. Int. J. Environ. Res. Public Health.

[20] Sugiyama T., Leslie E., Giles-Corti B., Owen N. (2008). Associations of neighbourhood greenness with physical and mental health: Do walking, social coherence and local social interaction explain the relationships?. J. Epidemiol. Community Health.

[21] Corazza M.V., Di Mascio P., Moretti L. (2016). Managing sidewalk pavement maintenance: A case study to increase pedestrian safety. J. Traffic Transp. Eng..

[22] Ewing R., Schroeer W., Greene W. (2004). School location and student travel. Transp. Res. Rec..

[23] Davidson K.K., Lawson C.T. (2006). Do attributes in the physical environment influence children’s physical activity? A review of the literature. Int. J. Behav. Nutr. Phys. Act..

[24] Gomez J.E., Johnson B.A., Selva M., Sallis J. (2004). Violent crime and outdoor physical activity among inner-city youth. Prev. Med..

[25] Rahman M.T. (2016). Detection of land use/land cover changes and urban sprawl in Al-Khobar, Saudi Arabia: An analysis of multi-temporal remote sensing data. ISPRS Int. J. Geo-Inf..

[26] Aina Y.A., Merwe J.H.V., Alshuwaikhat H.M. Urban Spatial Growth and Land Use Change in Riyadh: Comparing Spectral Angle Mapping and Band Ratioing Techniques. Proceedings of the Academic Track of the 2008 Free and Open Source Software for Geospatial (FOSS4G) Conference, Incorporating the GISSA 2008 Conference.

[27] Rahman M.T., Aldosary A., Nahiduzzaman K.M., Reza I. (2016). Vulnerability of flash flooding in Riyadh, Saudi Arabia. Nat. Hazards.

[28] Alrashed F., Asif M. (2014). Trends in Residential Energy Consumption in Saudi Arabia with Particular Reference to the Eastern Province. J. Sustain. Dev. Energy, Water Environ. Syst..

[29] Alhubashi H.H. (2012). Housing Sector in Saudi Arabia: A Study of Challenge and Opportunities of Homeownership for the Middle and Low Income.

[30] Rahman M.T., Aldosary A.S., Mortoja M.G. (2017). Modeling Future Land Cover Changes and Their Effects on the Land Surface Temperatures in the Saudi Arabian Eastern Coastal City of Dammam. Land.

[31] Dehwah A., Asif M., Rahman M.T. (2018). Prospects of PV application in unregulated building rooftops in developing countries: A perspective from Saudi Arabia. Energy Build..

[32] Rahman M.T. (2018). Examining and modelling the determinants of the rising land surface temperatures in arabian desert cities: An example from Riyadh, Saudi Arabia. J. Settlements Spat. Plan..

[33] Aljoufie M., Zuidgeest M., Brussel M., Maarseveen M.V. (2013). Spatial–temporal analysis of urban growth and transportation in Jeddah City, Saudi Arabia. Cities.

[34] Algadhi S., Mufti R., Malick D. (2002). Estimating the Total Number of Vehicles Active on the Road in Saudi Arabia. J. King Abdulaziz Univ. Eng. Sci..

[35] Almahamood M., Scharnhorst E., Carstensen T.A., Jorgenses G., Schulze O. (2017). Mapping the gendered city: Investigating the socio-cultural influence on the practice of walking and the meaning of walkscapes among young Saudi adults in Riyadh. J. Urban Des..

[36] Al-Ghamdi A.S. (2002). Pedestrian-vehicle crashes and analytical techniques for stratified contingency tables. Accid. Anal. Prev..

[37] Shokry H., Maksoud R. Improving Walkability within Existing Urban Design. Proceedings of the 3rd International Conference on Liveable Cities—A Joint Conference with International Conference on Engineering, Innovation & Technology (ICLC2015 & EIT2015).

[38] Dhahran Population. http://population.city/saudi-arabia/dhahran/.

[39] World Weather and Climate Information Average Weather in Al-Khobar, Saudi Arabia. http://www.weather-and-climate.com/average-monthly-Rainfall-Temperature-Sunshine,al-khobar,Saudi-Arabia.

[40] Myint S.W. (2008). An exploration of spatial dispersion, pattern, and association of socio-economic functional units in an urban system. Appl. Geogr..

[41] Chebat J.C., Gélinas-Chebat C., Therrien K. (2008). Gender-related wayfinding time of mall shoppers. J. Bus. Res..

[42] McLeod K. (2013). WHERE WE RIDE: Analysis of Bicycling in American Cities.

[43] Nabielek K., Hamers D., Evers D. (2016). Cities in Europe: Facts and Figures on Cities and Urban Areas.

[44] Friedman L. Here Are The States Where The Most People Walk Or Bike To Work. http://www.businessinsider.com/here-are-the-states-where-the-most-people-walk-or-bike-to-work-2014-7.

[45] Kitchen P., Williams A., Chowhan J. (2011). Walking to work in Canada: Health benefits, socio-economic characteristics and urban-regional variations. BMC Public Health.

[46] Cravo V.S., Cohen J.E. The impact of weather on transit revenue in New York City. Proceedings of the Proceedings of the 88th Annual Meeting of the Transportation Research Board, Transportation Research Board of National Academies.

[47] Clarke P., Hirsch J.A., Melendez R., Winters M., Gould J.S., Ashe M., Furst S., McKay H. (2017). Snow and Rain Modify Neighbourhood Walkability for Older Adults. Can. J. Aging.

[48] Yang Y., Roux A.V., Auchincloss A.H., Rodriguez D.A., Brown D.G. (2012). Exploring walking differences by socioeconomic status using a spatial agent-based model. Heal. Place.

[49] Laker L. Where is the World’s Most Walkable City?. https://www.theguardian.com/cities/2017/sep/12/walkable-city-worlds-most-new-york-melbourne-fes-el-bali.

[50] Budds D. The Hidden Inequality Of America’s Street Design. https://www.fastcodesign.com/3067055/the-hidden-inequality-of-americas-street-design.

[51] Ovstedal L., Ryeng E.O. Understanding pedestrian comfort in European Cities: How to improve walking conditions?. Proceedings of the European Transport Conference.

[52] Shaaban K., Muley D., Elnashar D. (2017). Evaluating the effect of seasonal variations on walking behaviour in a hot weather country using logistic regression. Int. J. Urban Sci..

